# THE HEALTH DISPARITIES AMONG OLDER ADULTS AGING IN PLACE: A RURAL-URBAN COMPARISON IN KOREA

**DOI:** 10.1093/geroni/igae098.3743

**Published:** 2024-12-31

**Authors:** Mi Sun Choi, Hyunmin Lee

**Affiliations:** Silla University, Busan, Republic of Korea; University of Seoul, Seoul, Republic of Korea

## Abstract

Aging in place (AIP) has emerged as a pivotal focus of welfare policies for the aging population in Korea, with health being a crucial determinant of AIP success. Regional variations can significantly impact the health of older adults who choose to remain in their communities. This study aims to elucidate the differences in factors affecting the health of older adults who continue to reside in their communities between urban and rural areas. Utilizing the ecological model of aging and the push and pull framework, we performed a multiple regression analysis on data from 647 older adults (aged 65 and above) from the Korean Welfare Panel Study, all of whom have lived in the same residence since the first wave in 2006. The analysis identified that, for both urban (n=342) and rural (n=305) older adults, work ability, levels of depression, and housing satisfaction significantly affect subjective health status. Specifically, for urban residents, male gender, higher income, compliance with minimum housing standards, and fewer outpatient visits were positively associated with health perception. The results show differences in health characteristics between urban and rural AIP older adults. In urban settings, both subjective perceptions and the physical housing environment play substantial roles in health, whereas, in rural areas, subjective perceptions are more pronounced. To promote health equity among AIP older adults, urban areas may benefit from targeted housing improvement initiatives, whereas enhancing the living environment in rural communities is essential for supporting the well-being of the aging population in these areas.

